# Predicting population genetic change in an autocorrelated random environment: Insights from a large automated experiment

**DOI:** 10.1371/journal.pgen.1009611

**Published:** 2021-06-23

**Authors:** Marie Rescan, Daphné Grulois, Enrique Ortega Aboud, Pierre de Villemereuil, Luis-Miguel Chevin

**Affiliations:** 1 CEFE, CNRS, Université de Montpellier, Université Paul Valéry Montpellier 3, EPHE, IRD, Montpellier, France; 2 Université Perpignan Via Domitia, Centre de Formation et de Recherche sur les Environnements Méditerranéens, UMR 5110, Perpignan, France; 3 CNRS, Centre de Formation et de Recherche sur les Environnements Méditerranéens, UMR 5110, Perpignan, France; 4 Institut de Systématique, Evolution, Biodiversité (ISYEB), Ecole Pratique des Hautes Etudes PSL, MNHN, CNRS, Sorbonne Université, Université des Antilles, Paris, France; University of Western Ontario, CANADA

## Abstract

Most natural environments exhibit a substantial component of random variation, with a degree of temporal autocorrelation that defines the color of environmental noise. Such environmental fluctuations cause random fluctuations in natural selection, affecting the predictability of evolution. But despite long-standing theoretical interest in population genetics in stochastic environments, there is a dearth of empirical estimation of underlying parameters of this theory. More importantly, it is still an open question whether evolution in fluctuating environments can be predicted indirectly using simpler measures, which combine environmental time series with population estimates in constant environments. Here we address these questions by using an automated experimental evolution approach. We used a liquid-handling robot to expose over a hundred lines of the micro-alga *Dunaliella salina* to randomly fluctuating salinity over a continuous range, with controlled mean, variance, and autocorrelation. We then tracked the frequencies of two competing strains through amplicon sequencing of nuclear and choloroplastic barcode sequences. We show that the magnitude of environmental fluctuations (determined by their variance), but also their predictability (determined by their autocorrelation), had large impacts on the average selection coefficient. The variance in frequency change, which quantifies randomness in population genetics, was substantially higher in a fluctuating environment. The reaction norm of selection coefficients against constant salinity yielded accurate predictions for the mean selection coefficient in a fluctuating environment. This selection reaction norm was in turn well predicted by environmental tolerance curves, with population growth rate against salinity. However, both the selection reaction norm and tolerance curves underestimated the variance in selection caused by random environmental fluctuations. Overall, our results provide exceptional insights into the prospects for understanding and predicting genetic evolution in randomly fluctuating environments.

## Author summary

Being able to predict evolution under natural selection is important for many applied fields of biology, ranging from agriculture to medicine or conservation. However, this endeavor is complicated by factors that inherently limit our ability to predict the future, such as random fluctuations in the environment. Population genetic theory indicates that probabilistic predictions can still be made in this context, but the extent to which this holds empirically, and whether these predictions can be based on simple measurements, are still open questions. Making progress on answering these questions can be achieved by capitalizing on experiments where the environment is precisely controlled over many generations. Here, we used a pipetting robot to generate random time series of salinities with controlled patterns of fluctuations, which we imposed on a microalga, *Dunaliella salina*. Tracking the frequencies of two genotypes in a mixture by sequencing two short barcode sequences, we were able to show how patterns of fluctuating selection relate to the fluctuating environment. Interestingly, parts of these responses, but not all, could be predicted by simpler measurements in constant environments, allowing precise characterization the limits and prospects for predicting evolution in fluctuating environments.

## Introduction

To what extent is evolution predictable? This question has received considerable interest from evolutionary biologists, and has become increasingly quantitative as relevant data have accumulated. Original qualitative arguments about contingency versus necessity [1] or replaying life’s tape [2] have been replaced by more detailed empirical and theoretical investigations on the rate of parallel genetic evolution in replicate lines or populations exposed to similar selective pressures [3–7]. Only recently has predictability in the *dynamics* (rather than outcome) of evolutionary change gained more prominence [8–11]. This question is indeed of crucial importance for many applications of evolution where the rate of change matters more than the end result, including evolutionary rescue, pest control, antibiotic resistance, and all contexts where strong eco-evolutionary dynamics involve a race between adaptation and population growth or decline [12–16].

A major factor that may alter the predictability of evolution is environmental stochasticity [17,18]. Most natural environments exhibit random fluctuations—also known as stochastic noise—characterized by their variance, which determines their magnitude, and autocorrelation (or color in power spectrum [19,20]), which determines their predictability. Such environmental noise causes randomly fluctuating selection at the genetic and phenotypic levels, which may reduce the predictability of evolution in a number of ways [17,21,22]. First, unaccounted sources of environmental variability (micro-environmental variation) can increase noise in frequency dynamics, thus reducing the precision of selection estimates [23]. And second, even if the environment were perfectly known at a given time, its future would still be uncertain if it fluctuates randomly. Environmental stochasticity thus contributes to chance in evolutionary trajectories, causing allele frequencies to undergo random walks, similarly to genetic drift caused by the finiteness of populations [24–30].

Despite their randomness, stochastic evolutionary dynamics can still be predicted in a probabilistic sense, provided we are able to accurately model them with few parameters. For instance, diffusion approximations (routinely employed to analyze genetic drift [31]) use the magnitude of short-term variance in frequency change conditional on the current frequency (so-called infinitesimal variance [32]) to predict the cumulative influence of stochasticity on allelic frequencies in the long run. For genetic drift, this stochasticity parameter is inversely proportional to the effective population size [31]. Here we wish to measure analog parameters for environmental stochasticity, another major source of chance in evolution [18].

In theoretical models, population genetics in stochastic environments are usually parameterized by the distribution (mean, variance, autocorrelation) of selection coefficients over time, which drives the evolutionary dynamics in this context [24,28,30,33,34]. For instance, the stochastic variance in selection coefficients over one generation (or infinitesimal time step in continuous time) can be used to predict evolutionary outcomes over multiple generations, such as probabilities of fixation [27,29] or expected heterozygosities [28], analogously to the influence of effective population size for genetic drift. However, while the demographic consequences of the magnitude and autocorrelation of environmental variations have been experimentally explored [35–38], and evolutionary experiments have been performed under randomly changing environments [39–43], we are not aware of attempts to measure the stochastic variance of population genetic change under conditions where patterns of random environmental fluctuations have been experimentally manipulated.

**Fig 1:**
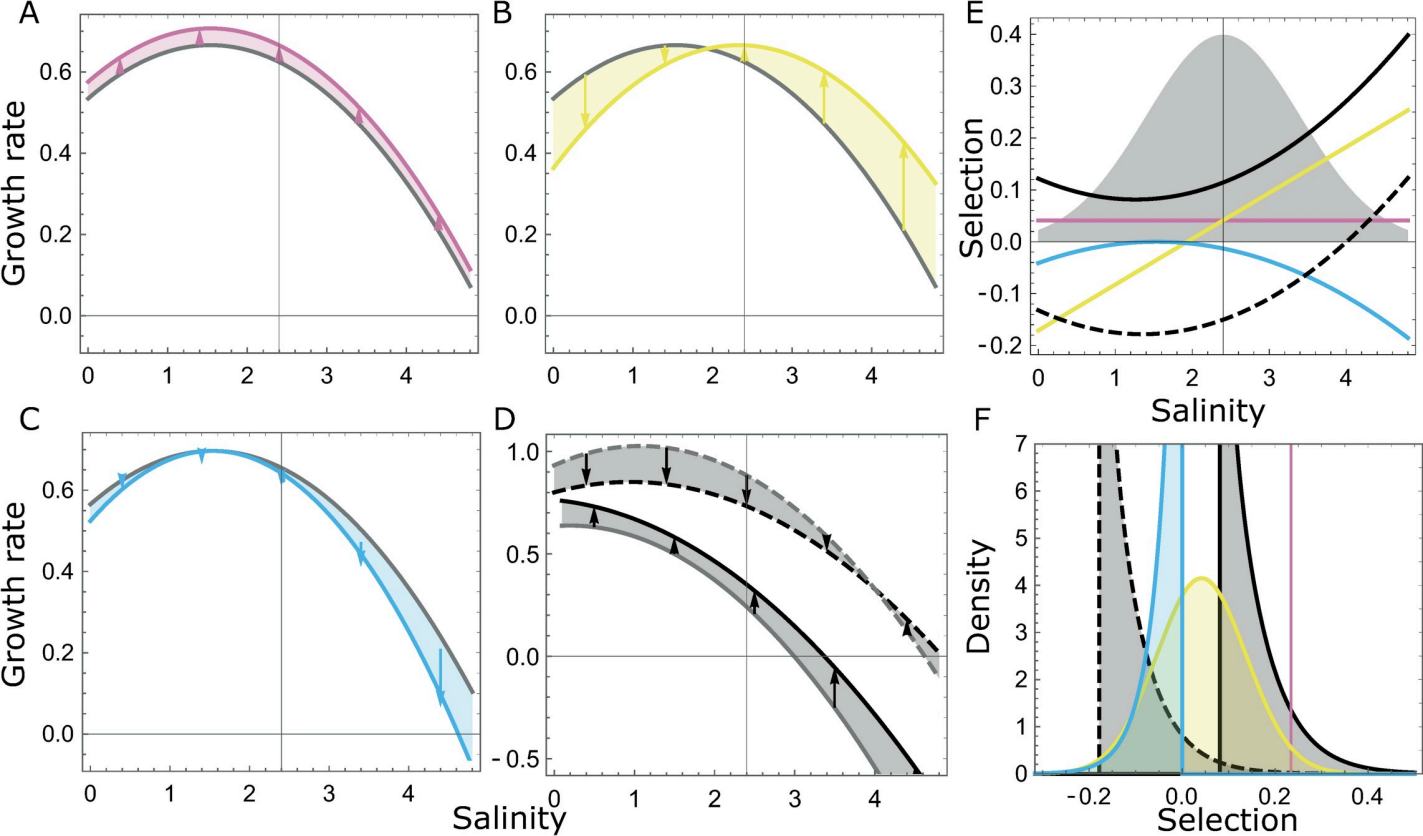
Relationship between tolerance curves and selection across environments. Panels a-d represent tolerance curves, with absolute fitness (population growth) in isolation plotted against the environment (here, salinity). In panels a, b and c, the tolerance curve of a reference strain (in gray) is contrasted to that of an alternative genotype or mutant (in color), which varies in only one parameter of the tolerance curve: maximal growth (A), salinity optimum (B), or niche width/breadth (C). Selection for the mutant equals the difference in growth rates between strains (shown as arrows) if fitness is density- and frequency-independent. In addition, the past environment may influence the current tolerance curve of each strain, as illustrated in panel d, where the tolerance curve of the mutant (in black) and reference strain (in gray) vary depending on whether they were transferred from low (plain line, 0.5M) or high (dashed, 4M) salinity. Panel e shows the selection reaction norm for the mutant based on these tolerance curves (hence assuming density- and frequency-independent selection), using the same line types and colors as in panels a-d. The gray normal curve materializes the salinity distribution used in our fluctuating salinity experiment (rescaled vertically for graphical convenience). Panel f plots the resulting distribution of selection coefficients in the fluctuating environment, with line types and colors as in previous panels. Note that the distribution of selection coefficient is Gaussian if both strains have the same tolerance breadth (yellow), but can otherwise be highly skewed.

Furthermore, even though these parameters of fluctuating selection are the most directly relevant for evolutionary predictions, it would also be extremely useful to be able to project evolutionary change based on how *the environment itself* fluctuates. Indeed, measurements of selection are complex, time consuming, and costly (often involving substantial sequencing effort), while massive environmental time series can readily be obtained from e.g. the Intergovernmental Panel on Climate Change [44] or collected anew using automated devices such as temperature loggers (Thermochrons [45]). Whether or not these abundant environmental data can be used to project evolutionary change depends on our ability to predict fluctuating selection from a fluctuating environment. The answer to this question is however not straightforward, and several degrees of simplification can be envisioned. First, it may be possible to measure selection coefficients at a few constant values of the environment, producing a form of “selection reaction norm”, which could then be combined with the pattern of environmental fluctuations to project population genetic change. Going one step further, one may simply measure the population growth rates in isolation of all genotypes across environments, to estimate their environmental tolerance curves [37,46,47]. Since selection arises from the differential growths rates of genotypes in competition, changes in selection across environments could then be inferred from genetic differences in tolerance curves (Fig 1), without requiring any sequencing effort to identify genotypes in mixtures. However, the usefulness and limits of such mechanistic links between the environment and selection first need to be evaluated under controlled conditions. For instance, the selection reaction norm approach would be compromised if selection at a given time in a given environment depends not only on the current environment, but also on the sequence of environments a population was exposed to, because of a memory of past environments mediated by phenotypic plasticity [37,40,48]. Similarly, fitness in competition may not be predictable from growth rates in monoculture (as assumed by the tolerance curve approach), if specific interactions between genotypes cause selection to be frequency- or density-dependent [49,50].

To investigate how random environmental fluctuations translate into randomly fluctuating selection, and elucidate the predictability of these responses, we here used experimental evolution in the laboratory, a powerful approach for quantifying the influence of environmental drivers on evolutionary dynamics [51–53], and assess precision limits in the measurement of key evolutionary parameters [23]. We worked with the halotolerant micro-alga *Dunaliella salina*, which we exposed to randomly fluctuating salinities over a realistic continuous range (instead of the more common practice of switching the environment between low and high constant levels [39–41]). We used a liquid-handling robot to control the mean, variance, and autocorrelation of salinity over many replicate lines. We tracked the frequencies of standing genetic variants through time by Illumina amplicon sequencing of two natural DNA barcodes, as done with engineered barcodes in other studies (BarSeq, [54,55]). We have previously shown that the stochastic demography of these populations was well predicted by combining patterns of environmental variation with short-term salinity tolerance curves [37]. Here, we ask whether, and how, key parameters of stochastic fluctuating selection are influenced by parameters of environmental variation: How much variance in selection is caused by variance in the environment? Does environmental variation also affect the mean selection coefficient? And is there an influence of environmental autocorrelation − which determines the predictability of environmental fluctuations − on patterns of fluctuating selection? We finally ask whether population genetics in a stochastic environment can be predicted by combining environmental time series with simpler population measurements, such as selection reaction norms and tolerance curves.

## Results

### Tracking population genetics in an experimental stochastic environment

**Fig 2:**
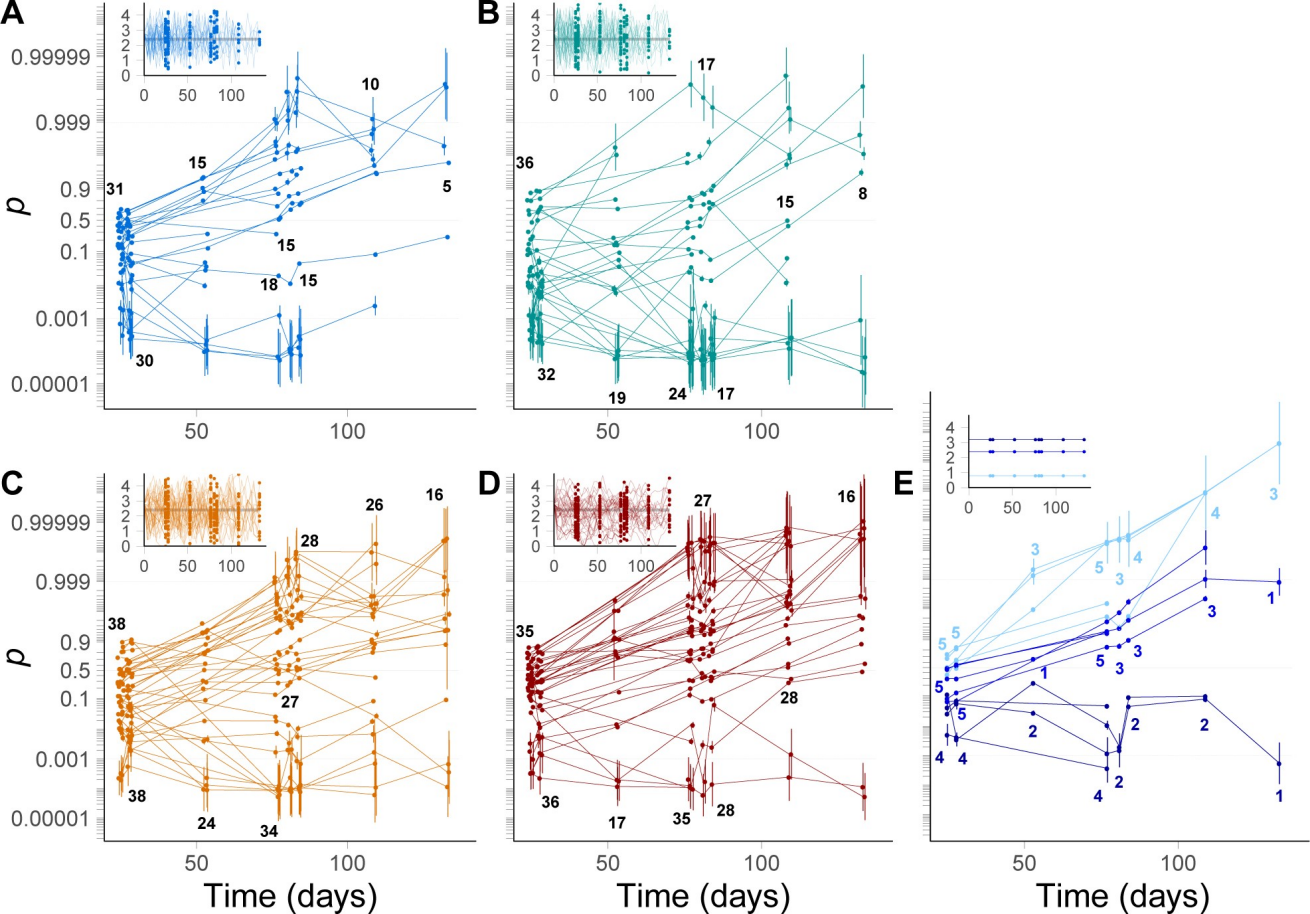
Frequency dynamics in fluctuating versus constant environments. Logit frequencies of all lines in fluctuating salinities (autocorrelation -0.5 (A, blue), 0 (B, green), 0.5 (C, orange), 0.9 (D, red)) and constant (E) salinities (from lighter to darker: 0.8M, 2.4M and 3.2M) are represented, with the corresponding salinity time series plotted in the insets. Frequencies were estimated as random parameters in the state-space logistic regression of choloroplastic and ITS2 marker sequences. Each line represents the frequency dynamics in one population, and numbers are the total number of populations sequenced in each salinity treatment and at each time point.

We followed the frequency of one strain (CCAP 19/15, hereafter denoted as C) of the microalgae *Dunaliella salina* competing in a mixture with another strain (CCAP 19/12, hereafter denoted as A) during 37 transfers (~100 generations), in constant or randomly varying salinity. We transferred each line twice a week (every 3 or 4 days), diluting 15% of the population of origin into fresh medium with controlled salinity, which differed across lines. Our treatments consisted of 138 independent fluctuating salinity time series with the same mean (*μ*_*E*_ = 2.4M NaCl) and variance $\left(\sigma_{E}^{2}=1\right)$ but four autocorrelation levels, from negative (*ρ* = −0.5) to highly positive (*ρ* = 0.9) (insets in Fig 2A–2D). We also had three constant treatments with salinity fixed to 0.8, 2.4 or 3.2M NaCl (insets in Fig 2E), with 4 to 5 replicates for each constant salinity. We sampled these populations at evenly spaced time points along the experiment, as well as at a few successive transfers (at steps 6–7, 20–21 and 21–22), so as to be able to connect short-term selection responses to long-term evolution in a fluctuating environment. We used Illumina amplicon sequencing of two barcode sequences (one nuclear and one choloroplastic) to track the frequency dynamics of strain C within and across lines under these different treatments (Fig 2). Some lines went extinct over the course of the experiment [37], so the sample size over which we investigated fluctuating selection decreased over time (Fig 2).

We inferred fluctuations in strain C frequency based on the combined number of Illumina reads at the ITS2 nuclear and the chloroplast locus, using a state-space model. Such a model treats the true stochastic frequency dynamics as an underlying, unobserved process. Conditional on the state of this process, the number of counts at the ITS2 and the chloroplast locus are then treated as two independent, binomially distributed observations. We used this model to analyze how the mean, variance, and autocorrelation of salinity influenced strain frequency dynamics. We assumed that the true logit allelic frequency $\Psi =ln\;\left(\frac{p}{1-p}\right)$ (with *p* the frequenecy of strain C) follows a Gaussian process over time, with mean $\bar{\Psi_{t}}$ and variance var(*Ψ*_*t*_) at time *t* given by

$$\begin{array}{c} \bar{\Psi_{t}}=\bar{\Psi_{0}}+(\theta_{0}+\theta_{1}\;\mu_{E}+\theta_{2}\;\mu_{E}^{2}+\theta_{3}\;\sigma_{E}^{2}+\theta_{4}\;\rho_{E}^{2})t\\ \mathrm{var}(\Psi_{t})=\mathrm{var}(\Psi_{0})+\frac{25}{7}t\{\begin{array}{c} \sigma_{0}^{2}\;\mathrm{if}\;\sigma_{E}^{2}=0\\ \sigma_{1}^{2}\;\mathrm{if}\;\sigma_{E}^{2}\neq 0 \end{array} \end{array}$$ (1)

The term in parentheses in the first line is the mean selection coefficient $\bar{s}$, which depends on the mean *μ*_*E*_, variance $\sigma_{E}^{2}$, and predictability $\rho_{E}^{2}$ of salinity. We assumed that the variance of selection (second line) depends on whether or not salinity fluctuates (that is, whether environmental variance is $\sigma_{E}^{2}=0$ or $\sigma_{E}^{2}\neq 0$). We did not test for an effect of the mean environment on the variance of selection because all fluctuating treatments had the same mean; models with effects of environmental autocorrelation on the variance of selection were tested but did not converge, see below. The coefficient 25/7 for the variance in Eq (1) corrects for the fact that salinity remains constant for a few days in between each transfer (S1 Appendix). We wrote an explicit joint probability density function in C++ and maximized its likelihood in R (v.3.5.2), using Laplace approximation implemented in the R package TMB [56]. More detail on the model is provided in the Methods. R and C++ codes are available from the Dryad digital repository [57].

### The average selection is reduced in fluctuating environments

**Fig 3:**
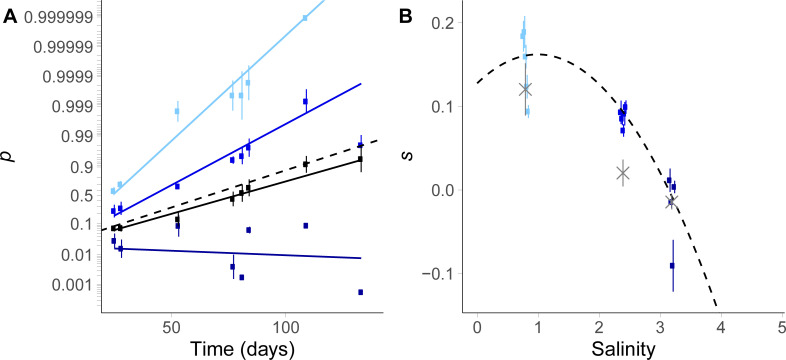
Mean selection in fluctuating versus constant environment. (A) Mean trajectories of logit allelic frequency over time. Solid lines represent logistic regression fits (Eqs (1) and (2)), while dots and error bars are time-specific estimates and bootstrapped standard deviations of logit frequencies, based on random factors in the state-space model. Black: fluctuating salinity (all autocorrelations treatments were pooled). Blue: constant salinities, from lighter to darker: 0.8M, 2.4M and 3.2M. The dashed line is the mean logit frequency dynamics as predicted from the selection reaction norm in panel b. (B) Selection reaction norm in constant environment. The selection coefficient for strain C is shown as dots and error bars for the logistic regression fit independently in each independent AC populations (colors match those in panel a). The dashed line represents the model that includes the influence of salinity on selection (selection reaction norm), and the gray crosses are the prediction from the difference in growth rates between strains A and C in monoculture (with error bars corresponding to the standard error).

The parameters of environmental fluctuations had a strong impact on the dynamics of population genetic change (as summarized in Table 1). The mean dynamics of logit-frequency (and therefore the mean selection coefficient $\bar{s}$) across replicates depended on the mean salinity, with larger positive selection for strain C at lower salinity (P < 10^−33^, Table 1). In addition, the mean selection coefficient was also strongly influenced by the temporal variance in salinity (P < 10^−18^). Pooling all data from fluctuating treatments (regardless of salinity autocorrelation), we found a lower advantage for strain C in stochastic environments, as compared to constant environments with the same mean (2.4M NaCl; compare black and middle blue line in Fig 3A).

**Table 1:** Effects of the temporal mean *μ*_*E*_, variance $\sigma_{E}^{2}$ and squared autocorrelation $\rho_{E}^{2}$ (or predictability) of the environment on the mean (Fixed effets) and variance (Random effects) of strain C logit frequency *ψ* (Estimates, Standard errors and P-values from Wald test). All terms representing an interaction with time *t* measure an effect of the environment on selection.

Fixed effects: $\bar{\psi_{t}}$			
	Estimate	Std. Error	Pr(>|W)
*t* (*θ*_0_ = *a*)	0.0927	0.00817	7.48E-30
*t*:*μ*_*E*_ (*θ*_1_ = *b*)	-0.0994	0.00852	8.41E-34
$t:\mu_{E}^{2}(\theta_{2}=c)$	-0.0353	0.00718	6.87E-07
$t:\sigma_{E}^{2}(\theta_{3})$	-0.0765	0.00864	7.68E-19
$t:\rho_{E}^{2}(\theta_{4})$	0.0882	0.0108	3.95E-16
$\bar{\psi_{0}}$	-3.80	0.266	8.97E-46
Random Effects: V(*ψ*_*t*_)			
*V*(*ψ*_0_)	4.90E-05	0.00409	9.90E-01
$t:[\sigma_{E}^{2}=0](\sigma_{0}^{2})$	0.0196	0.00427	4.26E-06
$t:[\sigma_{E}^{2}=1](\sigma_{1}^{2})$	0.129	0.00828	1.15E-54

To investigate to what extent this effect of environmental stochasticity on the mean selection coefficient can be predicted from first principles, we measured the direct influence of the environment on selection using a selection reaction norm in constant salinity (Fig 3B). We assumed a quadratic shape for selection as function of a constant environment,

$$s=a+b\;E+c\;E^{2}$$ (2)

where *E* is the (constant) environment, measured as deviation from the mean salinity. Coefficients *a*, *b* and *c* are directly given by *θ*_0_, *θ*_1_ and *θ*_2_ in Eq (1) when environmental variance and autocorrelation are set to 0. We found that in a constant environment, strain C is favored in the intermediate environment where salinity is 2.4M NaCl (*a* > 0, P < 10^−29^, see Table 1), but is less favored at higher salinity (*b* < 0, P < 10^−33^), with an advantage that vanishes towards 3M NaCl. In addition, there is a negative quadratic effect of constant salinity on selection (*c* < 0, P < 10^−6^), such that the salinity reaction norm is concave, with an optimum at an intermediate salinity (Fig 3B). For the reaction norm estimated by Eq (2), the maximal selection coefficient for strain C is 0.16, which occurs at an optimal salinity of 1.0M, and the breadth of the hump around the optimum (defined as $1/\sqrt{-c}$) is 5.5M (Fig 3B).

Assuming that selection is density- and frequency-independent, and is not affected by any memory of past environments ([37] and below), then selection coefficients measured over a range of constant environments (selection reaction norm) can be used to predict the dynamics of (logit) allele frequency in a fluctuating environment, by summing selection over all past environments experienced by both strains [24,49]. Combining the reaction norm in Eq (2) with the (nearly) normal distribution of salinities in our evolutionary experiment, the predicted mean selection coefficient is

$$\bar{s}=a+b\;\mu_{E}+c\;(\mu_{E}^{2}+\sigma_{E}^{2}).$$ (3)

Eq (3) highlights that in a stationary stochastic environment, and assuming that the selection reaction norm follows Eq (2), environmental variance affects the mean selection coefficient only if there is a quadratic effect of salinity on selection (*c*≠0). In particular when *c*<0, the mean selection coefficient in a fluctuating environment is reduced relative to a constant environment, by an amount $c\;\sigma_{E}^{2}$ that strikingly does not depend on what the mean environment is (but the mean environment still influences the probability to shift from negative to positive selection for a given environmental variance $\sigma_{E}^{2}$). In contrast when *c* = 0, Eq (3) predicts the same expected (logit) frequency dynamics in constant vs stochastic environments, if they have the same mean *μ*_*E*_. The expected logit strain mean frequency $\bar{\Psi_{t}}$ over time based on the quadratic selection reaction norm (Eq 3) is shown as dashed lines in Fig 3A, and matches very well the mean frequency dynamics estimated in fluctuating environments (solid black line in Fig 3A; P = 0.32 for the Wald test between selection estimate and reaction norm prediction). This indicates that the reduced expected selection coefficient in a fluctuating environment compared to a constant environment with same salinity mean likely arises from the concavity of the relationship between selection and the environment (a case of Jensen’s inequality).

### Environmental predictability also altered the expected frequency dynamics

**Fig 4:**
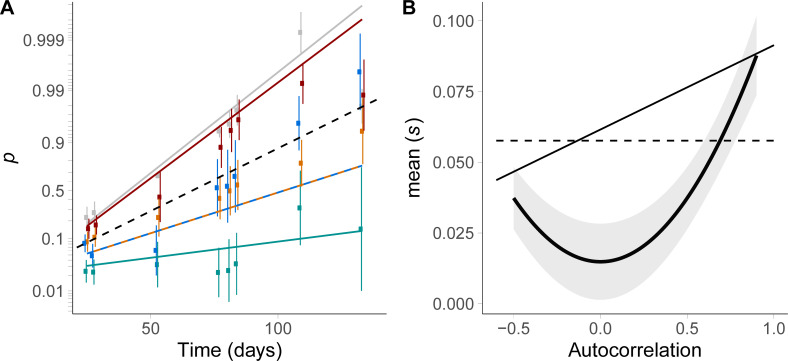
Influence of environmental autocorrelation on selection. (A) Frequency dynamics for treatments with different autocorrelations (-0.5 blue, 0 green, 0.5 orange, 0.9 red). Dots and error bars are the means and standard errors bootstrapped from the realized logit frequencies, and lines give the logistic state-space model predictions (Eqs (1) and (2)). Gray: constant environment with same mean salinity 2.4M. Dashed black line: prediction from the selection reaction norm built in constant environments (Eq (3)). (B) Influence of autocorrelation on mean selection. The black thick curve and ribbon show the mean selection coefficient $\bar{s}$ estimated by the logistic state-space regression, and its 95% confidence intervals computed with the delta method. Lines are the predictions from the univariate (dashed line, Eq (2)) and bivariate (solid line, Eq (5)) selection reaction norm.

Patterns of stochastic environmental fluctuations are not only characterized by their variance, which relates to their magnitude, but also by their autocorrelation, which determines the pattern of salinity transitions. This pattern has a major impact on the population dynamics of each strain in isolation in this species [37], and may therefore also alter selection among them in a stochastic environment. We found that environmental autocorrelation had a substantial influence on mean selection (P < 10^−15^, LR test between the regression described by Eq (1) and the same model without autocorrelation effect on mean selection). The mean selection for strain C in stochastic environments significantly increased with increasing squared autocorrelation $\rho_{E}^{2}$ of salinity (Fig 4), a measure of their predictability. The predictions from Eq (3) matched well the dynamics under intermediate predictability (autocorrelation *ρ* = ±0.5, compare orange and blue dots and lines to the dashed black line prediction in Fig 4), while in the more autocorrelated environment where transitions are smallest (*ρ* = 0.9), the mean selection coefficient exceeded the predictions from Eq (3), and was instead very close to that under constant salinity 2.4M (mean of the fluctuating treatments; compare red and gray dots and lines in Fig 4). Importantly, we found no significant difference between autocorrelation 0.5 and -0.5 (P = 0.50, likelihood ratio test between and the model described by Eq (1) and the full model that also includes an effect of unsquared autocorrelation *ρ*_*E*_, Eq 12 in the Methods). This suggests that allele frequency dynamics did not respond to the magnitude of salinity transitions, controlled by their temporal autocorrelation, but rather to their predictability, as determined by the squared autocorrelation.

Because temporal autocorrelation does not change the stationary distribution of environmental values, but only the similarity between two successive environments, the significant effect of environmental predictability on mean selection must involve an effect of past environment on current selection. In fact, the salinity reaction norm in Eq (2), which only depends on current environment, predicts no influence of environmental autocorrelation on mean selection (dashed line in Fig 4B).

In order to capture the influence of past environment on selection, we used the known past and current salinities *E*_t-1_ and *E*_t_ from successive transfers (at steps 6–7, 20–21 and 21–22 of the experiment) as predictors for selection in the experiment with fluctuating salinity. Within the logistic framework described by Eq (1), this led to

$$\Psi_{t}∼N(\Psi_{t-1}+t\;s(E_{t-1},E_{t}),{t^{2}\;\sigma }_{s}^{2}),$$ (4)

where *Ψ*_*t*−1_ and *Ψ*_*t*_ are the logit frequency before and after each transfer (duration *t* = 3 or 4 days), and *s*(*E*_t-1_, *E*_t_) models a bivariate selection reaction norm where selection coefficients depend on the current and previous salinity (see S1 Table and S1 Fig):

$$s(E_{t-1},E_{t})=a^{\prime }+b^{\prime }E_{t}+c^{\prime }E_{t}^{2}+d\;E_{t-1}+e\;E_{t-1}^{2}+f\;E_{t}E_{t-1}.$$ (5)

We found a significant effect of current (P = 2.7 10^−2^, LR test between a model considering only current salinities and a model with constant selection) and past salinities (P = 5.0 10^−3^, LR test between a model with a bivariate vs a univariate selection reaction norm) on selection coefficients. In addition, population density and allele C frequency in first environment *E*_t-1_ were variable in our data (density ranging from 10^3^ to 6,5 10^5^ cells.mL^-1^, and frequency from 0.9% to 99.7%, respectively). We injected terms for the influences of population density and strain frequency on selection in Eq (5), but found no signal of density- (P = 0.55) nor frequency-dependent selection (P = 0.08). Combining Eq (5) with the binormal distribution of salinities at two successive transfers, we found that the predicted mean selection coefficient was higher in more autocorrelated environment as observed in our experiment, but was largely overestimated in unpredictable environments with *ρ* = 0 (thin line vs thick line and ribbon in Fig 4B).

### Selection reaction norm under-predicts selection variance

**Fig 5:**
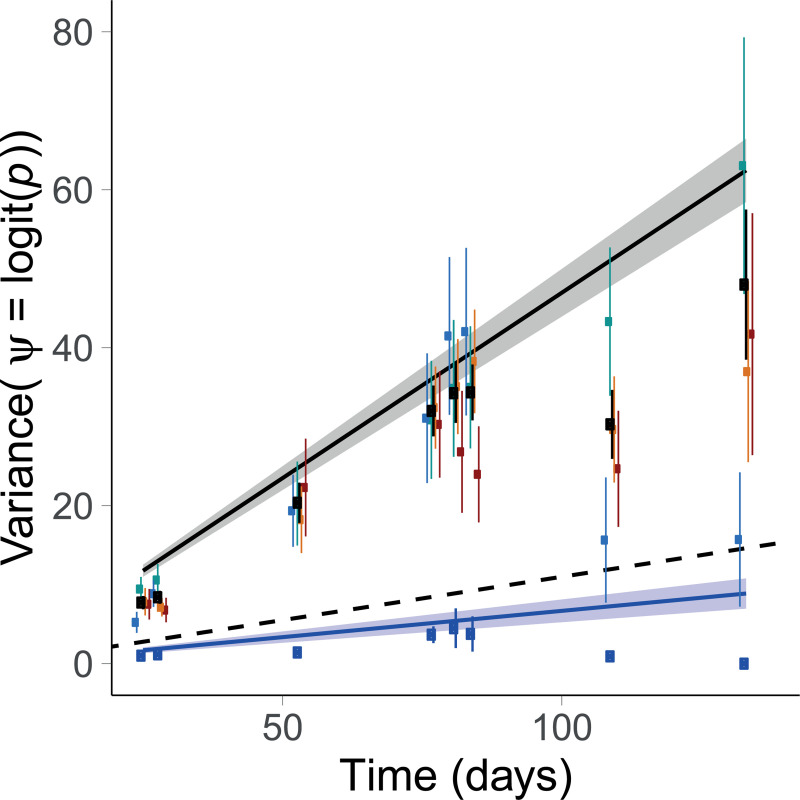
Frequency variance in constant versus fluctuating environments. Solid lines and ribbons represent the logistic regression estimation and standard errors (Eq (1)) in constant (blue) and stochastic (black) environments, while the dashed line is the prediction from the univariate selection reaction norm (Eq (2)). Dots and error bars are the mean and bootstrapped standard deviation of the realized logit frequencies estimated as random factors in the state-space model. Variance was assume to be the same within all constant treatments (blue) and all fluctuating treatments (black), and bootstraps were performed after subtracting the mean frequency. Bootstrapped variance measured in each autocorrelation treatment is also represented (-0.5 blue, 0 green, 0.5 orange, 0.9 red).

The variance of strain C frequency increased substantially faster over time in a stochastic environment as compared to a constant environment (P < 10^−33^, Fig 5), consistent with predictions from theoretical population genetics [27,29,30,34,58]. The variance in selection $\sigma_{s}^{2}$ was over seven times higher in stochastic as compared to constant treatments. We nevertheless observed a significant increase in allele frequency variance ($\sigma_{s}^{2}>0$) even in constant salinities, which may result from genetic drift and/or from micro-environmental variation impossible to control for (slight light and temperature heterogeneity in the incubator).

From the univariate selection reaction norm in Eq (2), the expected variance of selection coefficients caused by fluctuating selection is:

$$var(s)=\sigma_{E}^{2}(b^{2}+4\;b\;c\;\mu_{E}+2c^{2}(2\mu_{E}+\sigma_{E}^{2}))$$ (6)

This predicts a selection variance $\sigma_{s}^{2}=0.012$, which is about one order of magnitude below the variance measured in fluctuating environment (0.13). Selection variance given by the logistic regression was the sum of selection variance attributable to salinity fluctuations, predicted by Eq (3), plus a residual variance corresponding to that measured in a constant environments ($\sigma_{0}^{2}$ in Eq (1)). We injected this predicted variance in Eq (1) to compute the expected variance of logit transformed allelic frequency. However, even after adding the selection variance measured in constant environments, predictions from the selection reaction norm remained more than 4 times lower than the variance estimated in the logistic regression (Fig 5).

Eq (6) predicts that the variance in selection coefficients increases with the environmental variance, but is not affected by autocorrelation, if selection only depends on the current environment. To relax the latter assumption, we obtained the selection variance predicted by the bivariate selection reaction norm in Eq (5), which also includes an influence of the previous environment. This barely changed the prediction for selection variance, which was still drastically below the variance estimated in constant environments (between 0.025 and 0.03 when summed to the selection variance in constant environment).

### Tolerance curves accurately predict selection only in constant environments

In a population comprising two genotypes, the selection coefficient can be translated into a difference between the *per-capita* population growth rates of genotypes in competition [31,49],

$$s=\frac{d}{dt}ln\left(\frac{p}{1-p}\right)=\frac{d}{dt}ln\left(\frac{C}{A}\right)=r_{C}-r_{A}$$ (7)

where *C* and *A* are the population densities of the two genotypes, and *r*_*C*_ and *r*_*A*_ their *per-capita* growth rates (equal to the slope of the population growth curve on the log scale). If selection is density-independent (meaning that the *per-capita* growth rates of both genotypes respond identically to population density), and there are no interactions between strains (no frequency dependence), then the intrinsic growth rates of both strains, each measured in monoculture, can directly be used to predict the outcome of selection in a given environment [49]. When this holds, selection in a fluctuating environment can be predicted by comparing the tolerance curves of both strains, which describes how their intrinsic rates of increase measured in monoculture change with the environment (Fig 1). In particular, genotypes with the same tolerance breadth (implying similar plasticity [47,59]) would be expected to have a linear selection reaction norm, leading to a normal distribution of selection coefficients if the environment follows a Gaussian process [24]. Conversely, any curvature in the selection reaction norm in Eq (2) would be interpreted as a difference in tolerance breadths of the strains, reflecting differences in the plasticity of underlying traits influencing fitness across environments [24,60], and leading to skewed distribution of selection coefficients (Fig 1).

In our fluctuating treatments, selection measured over one salinity transfer was not correlated with strain differences in their per-capita population growth rates, as estimated by Rescan et al. [37] (S2 Fig). This is not necessarily surprising, given that most variance in our stochastic treatments was not explained by our salinity tolerance curves. By contrast, selection estimated over 37 transfers in constant environments was in a good agreement with differences between the per-capita growth rates of strains in monoculture (non-significantly different at salinity = 0.8 and 3.2M, but slightly below the selection measured in competition at 2.4M, P <10^−6^, Wald test; see gray crosses in Fig 3B). A notable discrepancy was that the growth rates in monoculture predicted a linear selection reaction norm, because the two strains have similar salinity tolerance breadths (95% bootstrapped confidence interval: [–6 10^–3^, 2 10^–2^], from 1000 simulations and fit of a quadratic reaction norm). Based on this, we would predict that the mean allelic frequency does not differ between stochastic and constant environments (Eq 3), contrary to what we observed in our experiment.

## Discussion

### Fluctuating environments as a source of stochasticity

The extent to which evolution in a stochastic environment can be predicted is still largely an open question. The intense selectionist vs neutralist debate in the second half of the XXth century somewhat crystallized on the relative roles of drift vs fluctuating selection as sources of chance in population genetics [25,61–63]. This question finds ramifications in todays’ science [64], but its answer ultimately rests on empirical quantitative evidence: it depends on how the variance in selection compares to the reciprocal of the effective population size. Using over 150 replicate lines of two competing strains exposed to fluctuating salinities with controlled mean, variance, and autocorrelation, we experimentally quantified the influence of random environmental fluctuations on stochasticity in selection. We found that selection variance was seven times higher in fluctuating than in constant salinities (Fig 5), revealing an important contribution of environmental stochasticity to evolution in our experiment. This variance was much higher than predicted based on simple responses to salinity in a constant environment. A possible reason is that the fluctuating environment, by causing dramatic changes in population size [37], also led to increased genetic drift.

Beyond the randomness in evolutionary trajectories, one of the emerging debates in the early literature on fluctuating selection regarded whether or not stochastic fluctuations influence the expected strength of selection and mean evolutionary trajectory. Some models found that variance in selection coefficients influences the mean trajectory [30,34], and others that it does not [28,33], depending notably on the role of density regulation in selection [28,65]. More recent work has re-explored this question by explicitly including a phenotype under selection for a moving optimum, and predicted that the variance of fluctuations should only influence the expected selection if genotypes differ in their phenotypic plasticity and environmental tolerance [24]. Here, we found that the mean selection coefficient favoring strain C is substantially lower in a randomly fluctuating environment as compared to a constant environment with salinity fixed to the mean of the fluctuating treatment, as expected if the two strains differ in their plasticity levels [24] and/or are under density dependent selection. We found no signal for density dependence, whereas the concave shape of the selection reaction norm measured in constant environments suggests that our focal strain C actually has a narrower tolerance niche, consistent with a lower plasticity level [59].

### The role of plasticity in responses to environmental autocorrelation

In addition to the amplitude of environmental fluctuations, their autocorrelation pattern also affected the mean frequency trajectory, with higher selection for our focal strain in the more predictable environments (Fig 4). This suggests that selection depends not only on the current environment, but also on environmental transitions. Accordingly, taking the previous salinity into account improved the prediction for the expected frequency dynamics in temporally autocorrelated stochastic environment. This is consistent with the crucial effect of past salinity on population growth in *Dunaliella salina*, involving a transgenerational phenotypic memory mediated notably by the dynamics of glycerol content used as osmoprotectant. Theoretical studies predict that temporal autocorrelation should also affect the variance in evolutionary trajectories, either directly through its effect on autocorrelation in selection [24], or indirectly through its effect on population size variance [66], described as the inflationary effect [35]. Here, we lacked statistical power to fit different variances in slopes for each autocorrelation treatment, probably due to the loss of many populations and the quasi fixation of one strain in many others before the end of the experiment (Figs 2 and 5).

The frequency dynamics in fluctuating vs constant environment, and the selection reaction norm measured in constant environments, both point towards strain C being less plastic than its competitor. Finding that it is more advantaged in more predictable environments (Fig 4) therefore seems at odds with the theoretical prediction that higher levels of plasticity should be favored in more predictable environments [47,48,67–70]. A possible explanation could be that a trade-off exists between salt tolerance and reproduction in our *Dunaliella salina* strains. Indeed, strain C has a higher maximal growth rate than strain A, but the latter is able to grow at higher salinities. It has been shown both theoretically and experimentally that under such tradeoffs, white noise enhances the evolution of stress tolerance compared to more autocorrelated environments [41].

In a previously published study based on a follow-up to the present experiment over several months, we found that higher levels of morphological plasticity were maintained in pure lines of strains A and C that evolved in highly predictable environments [71], consistent with theoretical predictions. Although this seems partly at odds with our present results about the influence of temporal autocorrelation on the competition between strains A and C (in connection to the selection reaction norm that likely results from their differences in plasticity), these two studies nevertheless share striking features. They both revealed (i) an evolutionary outcome that is very similar between constant and highly predictable fluctuating environments, and (ii) a response that depends on environmental predictability (squared autocorrelation) rather than on temporal autocorrelation *per se*, with similar dynamics in negative and positive autocorrelation with the same absolute value (ρ = -0.5 vs 0.5). The latter is not trivially explained by the effect of salinity transitions on fitness, because the selection reaction norm with effects of past and current environments predicts a linear response of mean selection to autocorrelation (Fig 4B; see also [37] for the effect on growth rates of individual strains). Possible explanations may involve a more complex shape of the selection reaction norm (e.g. involving higher order terms such as $S_{t-1}\times S_{t}^{2}$, which would measure the effect of past salinity on the breadth of the current selection reaction norm), or an effect of the salinity two transfers in the past, with correlation *ρ*^2^ to the current salinity. Testing such hypotheses would require challengingly long time series of allelic frequency, but another line of evidence may come from analysis of the molecular mechanisms involved, such as transcriptomic and epigenomic responses to salinity transitions. In any case, understanding how environmental time series with opposite autocorrelation can lead to similar genetic dynamics is a necessary step towards forecasting the evolutionary consequences of fluctuating environments, especially since temperature predictability has changed over the last century [72–74], with either more positive or more negative autocorrelation depending on the location.

### The limits to predictability in evolution

Our study underlines the difficulty in predicting population genetic change in stochastic environments from more basic (and less labor intensive) data, such as tolerance curves or selection reaction norms measured in constant environments. Data on growth rates of individual genotypes are the easiest to obtain as they only require counts, and do not rely on sequencing effort. However here, they only gave a qualitatively acceptable prediction for selection, and only when measured in constant environment and in the long run. This suggests that interactions between strains cause their growth rate in competition to differ from that in monoculture, even though we did not detect any clear evidence of frequency-dependent selection. Another challenge is that a substantial part of the variance in allelic frequency change was not explained by simple responses to salinity captured by our selection reaction norms (Fig 5). In addition, we found significant variance in frequency change also in constant environments, pointing to uncontrolled sources of stochasticity even in laboratory conditions, possibly involving micro-environmental variation or drift due to the random sampling of individuals (because of bottlenecks upon transfers [75] and/or the demographic consequences of the fluctuating environment [37,66]). Overall, our results thus indicate that randomly fluctuating environments can strongly shape the dynamics of population genetic change, but that deciphering and predicting these effects may require more detailed information than is provided by population growth or even selection in constant environments.

## Methods

### Evolutionary experiment

We exposed a mixture of two *Dunaliella salina* strains to fluctuating vs constant salinity, and tracked their frequencies through time by amplicon sequencing. Details of this long-term experiment were described in a previous study focused on demography [37], so we only summarize them briefly here. Populations of *Dunaliella salina* were initiated by mixing 50% of strain CCAP19/12 (denoted below as strain A) and 50% of strain CCAP19/15 (hereafter strain C), and exposed to constant or fluctuating salinities during 37 transfers (~100 generations). Populations were transferred twice a week by diluting 15% of the culture into 800 μL of fresh medium using a liquid-handling robot (Biomek NXP Span-8; Beckman Coulter). At each transfer, the target salinity was achieved by mixing the required volumes of hypo- ([NaCl] = 0 M) and hyper- ([NaCl] = 4.8 M) saline media, after accounting for dilution of the pre-transfer salinity. Populations in constant salinities were exposed to 0.8, 2.4 and 3.2M NaCl, with 5 replicates per salinity, while the fluctuating treatments consisted of 39 independent stochastic salinity time series (with the first replicated three times), for each of 4 temporal autocorrelation levels. Salinities were sampled from a first-order autoregressive process (AR1) with mean 2.4M, variance 1 and autocorrelation -0.5, 0, 0.5 and 0.9.

Populations were frozen after transfers 6, 7, 8, 14, 21, 22, 23, 30 and 37 for extraction and amplification of a chloroplast locus and the ITS2. Before extraction, *Dunaliella* cells were killed by adding 120 μL of 100% ethanol to the 480 μL of culture left for each populations after replication and demographic measures [37]. After careful mixing, plates were centrifuged 5 min at 6000 rpm, supernatant removed, 200 μL PBS added for conservation and samples resupended. Plates were frozen at -20°C until extraction.

### Data acquisition

#### Amplicon sequencing

Genomic DNA was extracted using Nucleospin plant II (Macherey-Nagel) following the manufacturer’s protocols. Population density varied along time and between lines in our experiment [37], and only the 1071 samples with cell density greater than 10^3^/mL were extracted (representing 93% of all AC populations that were not extinct by the time of the transfer).

All samples were amplified for the ITS2 segment of the ribosomal gene, and a chloroplast locus. The primer of ITS2 gene amplified a fragment of 200bp, by ITS2-for2 (^5’^-GCAGAATTCCGTGAATCATCAAATC-^3’^) and ITS2-rev2 (^5’^-GCGAGCGATAAGCTGCCTACCCAGTTG-^3’^). For the chloroplast locus, new primers specific of each strain were drawn from Whole Genome sequencing of the strains, in order to obtain one fragment of 200bp. The primer of the chloroplast locus amplified a fragment of 200bp, by Chloro53-for1 (^5’^- CGTTTATCCATATACGGG-^3’^) and Chloro53-rev2 (^5’^- CGCGCGAGTACCATCAGGACC-^3’^). Both loci were amplified separately for each sample, with their specific primers and the addition of specific sequences to anchor indexing primers: forward (^5’^TCGTCGGCAGCGTCAGATGTGTATAAGAGACAGYR- [primers of ITS 2]-^3’^) and reverse ^5’^GTCTCGTGGGCTCGGAGATGTGTATAAGAGACAGYR- [primers of ITS 2]-^3’^) for the ITS2, and Chloro-N (^5’^TCGTCGGCAGCGTC AGATGTGTATAAGAGACAGN- and—NN—and—NNN—[primer Chloro]^3’^) and reverse (^5’^GTCTCGTGGGCTCGGAGATGTGTATAAGAGACAGN—and—NN—and—NNN—[primer of Chloro]^3’^) for the chloroplast locus. PCRs were carried out in a final volume of 20 μl containing 0.05–0.1 μg/ml template DNA, Phusion High-Fidelity PCR Master Mix with HF Buffer 2X (ThermoScientific), 1 μM forward and reverse primers. PCR amplifications were conducted in Eppendorf Mastercycler ep gradients S thermal cycler under the following conditions: 1 min initial denaturation at 94°C, 35 cycles of 1 min at 94°C, 1 min at T_m_°C, 1 min at 72°C, and a final extension at 72°C for 5 min. The annealing temperatures (T_m_°C) were 55°C for the ITS2 and 70°C for the chloroplast locus. Negative controls were included in both the extraction and amplification steps. All amplicons were checked on the agarose gels (1%) and 859 positive samples (for both the ITS2 and the chloroplast locus) were send to the sequencer.

Sequencing was performed by the “Genseq” platform of LaBEX CEMEB, Montpellier. For each sample, PCR products for the ITS2 and chloroplast locus were mixed in the same tube in balanced quantity, and purified. All samples underwent an additional step of amplification with specific primers that included 9-bp long tags, to construct libraries for Illumina sequencing. Libraries were normalized, pooled, and sequenced on 1 run of the MiSeq Sequencer (Illumina, San Diego, CA) using the 2 × 150 bp MiSeq Reagent Kit v2.

The quality of reads was satisfying, with only few reads having quality below 20 (phred score, FastQC). We obtained 1718 demultiplexed paired-end fastq files, corresponding to the 859 samples. Fastq files of the chloroplast locus and ITS2 sequences were split with a bash script matching the ITS2 and chloroplast locus primers. Only exact matches where retained, rising the dataset quality. This produced unpaired reads that were repaired using repair.sh from BBMap 38.3254. Reads shorter than the length of the ITS2 segment were removed using trimmomatic55. Reads were finally merged using bbmerge-auto.sh from BBMap, with more than 90% of the reads merging successfully for all samples. The output quality was re-analyzed with FastQC and was satisfactory (phred score > 30). Samples with no reads left were removed, leaving 853 ITS2 and 842 chloroplast locus files, with an average of respectively 3407 and 1422 reads. A summary of the sampling plan at the different steps–extraction, sequencing, analysis–is given in S2 Table.

#### Strain identification

We aimed at estimating the frequencies of strains A and C in mixtures based on amplicon sequencing data. However, all the identified SNPs cannot be used for that purpose, because strains A and C (which are not isogenic) may have shared variants, and SNPs present in only one or few copies may be due to sequencing errors. In order to make efficient use of the sequencing data, we reduced all chloroplast and ITS2 reads to short haplotypes made of a succession of few SNPs in linkage disequilibrium that individually maximized the *F*_*ST*_ among pure, reference cultures of A and C. We also considered pure cultures of *Dunaliella salina* strain CCAP 19/18 used in the same experimental set up for another study [37], to be able detect potential cross contamination. For each SNP, we computed the global Nei *G*_*st*_ for multiple alleles [76]:

$$\begin{array}{lll} G_{st} & = & 1-H_{S}/H_{T}\\ & = & 1-\frac{1-\frac{1}{I}\sum_{j\in \{A,T,G,C,\emptyset \}}\sum_{i\in \{A,B,C\}}p_{ij}^{2}}{1-\sum_{j\in \{A,T,G,C,\emptyset \}}{\left(\frac{1}{I}\sum_{i\in \{A,B,C)}p_{ij}\right)}^{2}} \end{array}$$ (8)

where *H*_*S*_ is the mean expected heterozygosity within strain (that is, the probability that two haplotypes drawn from the same strain are different), *H*_*T*_ the expected total heterozygosity (probability that two haplotypes drawn from the full sample combining all strains are different), *p*_*ij*_ the frequency of base *j* (possibly A, T, G, C or ø) in strain *i*, and *I* = 3 the total number of strains. For the chloroplast locus, we kept all SNPs with *G*_*ST*_ *>* 0.8, resulting in haplotypes specific to the pure strains A and C. In our experimental populations with A and C mixed, more than 99.9% of the reads matched one of our reference haplotypes, and could thus be tagged accordingly as A or C. (The remaining reads were removed in subsequent analyses.) For the ITS2 locus, strains A and C were much less differentiated, and haplotypes were built using 3 SNPs displaying a G_ST_ > 0.2. In our experimental populations with A and C mixed, more than 99.9% of the reads matched two of these ITS2 haplotypes from our reference cultures, and the remaining reads were removed. The first haplotype was specific to strain A (and was thus tagged as A allele), while the other was shared by both strains but more common in strain C (present in 100% of the reference strain C, but only about 20% of reference strain A), so it was tagged as C allele.

#### Calibration

To validate and calibrate our estimates of strain frequencies based on relative number of Illumina reads, we mixed references cultures of strains A and C in 800 μL, with predefined relative frequencies 0, 5, 10, 20, 30, 40, 50, 60, 70, 80, 90 and 95 and 100% (where 0 and 100% are the pure cultures described just above), at density 10^5^ cells.mL^-1^, with two replicates each. Calibration samples were frozen, the chloroplast and ITS2 loci were amplified, sequenced, and all reads converted to short haplotypes as described above (S3 Fig). When analyzing this data, we noticed the presence of ITS2 alleles from the sister species *Dunaliella viridis* (more than 50% of the total number of ITS2 sequences in pure strains A and C), evidencing a contamination of our references strains. Such contamination was absent from all experimental populations, indicating either that contamination occurred after the end of the experiment (the reference populations were extracted 16 months afterwards), or that *D*. *viridis*, if initially present, disappeared rapidly before the 7^th^ transfer in our experiment. No *viridis* alleles were detected at the chloroplast locus where primers were specifically designed to amplify our strains, and we observed a correct match between observed and expected frequency (adjusted *r*^2^ = 0.89). Given that such correlation achieved in the presence of contaminants is extremely likely to hold in the absence of *viridis* contamination in the analysis of the experimental populations, we use the allele C frequency measured at the chloroplast locus as a direct proxy for strain C frequency. At the ITS2 locus, after removal of all *viridis* sequences, we found a perfect linear relationship between the frequencies of allele C *p*_ITS2_ and the strain C frequency *p* (*p*_*ITS*2_ = 0.19+0.79*p*, adjusted *r*^2^ > 0.99).

### State-space logistic regression

We estimated fluctuating selection by tracking the dynamics of the frequency *p* of strain C through time. Population genetic change under selection (especially fluctuating selection) is more conveniently analyzed on the logit scale, where responses to selection are additive over time [24,27,49,58]. The logit is also the canonical link function for a generalized linear model with binomial error (logistic regression), which is well-suited for population genetic measurements of selection [23].

Here we estimated strain frequencies by combining two sources of genetic information, from the ITS2 and the chloroplast locus. Our rationale was that if the two loci are in strong linkage disequilibrium − as confirmed by the strong linear relationship between allelic frequencies at both loci (*r*^2^ = 0.96), which remained unchanged over time (P = 0.27 for time effect on the regression slope of allele C frequency measured at the ITS2 against at the chloroplast locus) −, then using both as indicators of strain identity makes more efficient use of the data than performing simple, univariate logistic regression on each marker. We thus considered that the measured frequencies at the ITS2 and chloroplast loci were two observations (with error) of a true, unobserved strain frequency *p*. Formally, this corresponds to a state-space model [77], where the true dynamics of strain frequency is treated as an unobserved underlying process, while the observations (frequencies at the ITS2 and chloroplast locus) have errors that are mutually independent after conditioning by the underlying process. A state-space model is thus fully specified by the distributions of the errors and the underlying process. We wrote an explicit likelihood function in C++, and optimized it using the TMB package in R (v.3.5.2). R and C++ codes are available from Dryad digital repository [57].

#### Observation model

At the chloroplast locus, the number *n*_*i*,*t*_ of C sequences in population *i* at time *t* was assumed to follow a binomial distribution with parameters the strain frequency *p*_*i*,*t*_ and the total number of chloroplast sequences $N_{i,t}^{chloro}$. At the ITS2 locus, the number *m*_*i,t*_ of sequences C followed a similar binomial distribution, with a linear correction (with coefficients *α* and *β*) to account for the presence of a logit frequency *α* of allele C in strain A:

$$\begin{array}{c} n_{i,t}∼Bin(p_{i,t}={logit}^{-1}(\psi_{i,t}),N_{i,t}^{chloro})\\ m_{i,t}∼Bin(\alpha +\beta \;p_{i,t}=\alpha +\beta \;{logit}^{-1}(\psi_{i,t}),N_{i,t}^{ITS2}) \end{array}$$ (9)

#### Process model

In each population *i*, the dynamics of allele C frequency *p* are such that:

$$\psi_{i,t}=\ln\frac{p_{i,t}}{1-p_{i,t}}=\psi_{0}+\int_{0}^{t}s_{i,t}\;dt$$ (10)

where *ψ*_*i*,*t*_ is the logit frequency of allele C, and *s*_*i*,*t*_ the selection coefficient in population *i* at time *t*. In other words, selection coefficients are integrated/summed over time in their contribution to logit allelic frequency *ψ*.

Integration of the stochastic differential Eq (10) leads to the distribution of allele C logit frequency at time *t*. In particular, when *s*_*i*,*t*_ follows a Gaussian process, then logit frequency also has a Gaussian distribution, with mean and variance that increase linearly over time, with slopes $\bar{s}$ (the mean selection coefficient), and $\sigma_{s}^{2}$ (the variance of selection coefficients), respectively [25,34]. More generally, summing/integrating selection coefficients over time in Eq (10) should eventually lead to a normal distribution of *ψ*_*i*,*t*_ because of the central limit theorem, so we used a Gaussian process to model the dynamics of logit frequency,

$$\begin{array}{c} \Psi_{t}∼N(\bar{\Psi_{t}},var(\Psi_{t}))\\ \bar{\Psi_{t}}=\bar{\Psi_{0}}+\bar{s}\;t\\ var(\Psi_{t})=var(\Psi_{0})+\frac{25}{7}\sigma_{s}^{2}t \end{array}$$ (11)

The factor 25/7 in Eq (11) serves as a correction for the fact that selection does not fluctuate continuously over time, but instead remains constant during the time interval between our bi-weekly transfers, which affects estimation of the variance but not the mean of selection (S1 Appendix). We investigated the influence of our experimental treatments on these parameters of fluctuating selection, by including the environmental mean *μ*_*E*_, squared mean *μ*^*2*^_*E*_, variance $\sigma_{E}^{2}$, and predictability $\rho_{E}^{2}$ (where E is the deviation from 2.4M, the expected mean salinity of fluctuating treatments), as covariates for $\bar{s}$ and $\sigma_{s}^{2}$ in the regression in eq (11), leading to

$$\begin{array}{c} \bar{s}=\theta_{0}+{\theta_{1}\;\mu }_{E}+\theta_{2}\;\mu_{E}^{2}+{\theta_{3}\;\sigma }_{E}^{2}+\theta_{4}\;\rho_{E}^{2}+\theta_{5}\;\rho_{E}\\ \sigma_{s}^{2}=\left\{ \begin{array}{c} \sigma_{0}^{2}\;if\;\sigma_{E}^{2}=0\\ \sigma_{1}^{2}\;if\;\sigma_{E}^{2}=1 \end{array}\right. \end{array}$$ (12)

where *θ*_0_ is the selection coefficient in constant medium salinity 2.4M, and *θ*_1_ to *θ*_5_ are the coefficients associated with the mean, squared mean, variance, predictability and autocorrelation of the environment, respectively. We used likelihood ratio tests to select the environmental variables that best explained allele C frequency dynamics, leading to Eq (1) in the results. In particular, we tested models without effect of autocorrelation (*θ*_5_ = 0) and / or predictability (*θ*_4_ = 0) on mean selection. We also tried to quantify the effect of environmental autocorrelation on selection variance $\left(\sigma_{s}^{2}=\sigma_{1}^{2}+\theta_{6}\;\rho_{E}\;if\;\sigma_{E}^{2}=1\right)$, but this model could not converge. We did not test for an effect of the mean environment on the variance of selection because all fluctuating treatments had the same mean.

We then searched for finer, short-term mechanisms underlying these aggregate macroscopic effects of fluctuating selection, by focusing on frequency change over subsequent transfers with known salinities. We tested for effects on selection of current and previous salinity (following the reaction norms in Eqs 2 and 5), as well as initial frequency, and initial population density. For each transfer, frequencies both before and after selection were considered as random variables, and estimated from the observation model.

Combining the quadratic selection reaction norm in Eq (2), estimated over the long run in constant environments, with the normal distribution of environments in our fluctuating treatments, leads to analytically tractable distributions of predicted selection coefficients in a fluctuating environment. When the selection reaction norm is linear (c = 0), the distribution of selection coefficients becomes normal (Fig 1B, 1E and 1F), as expected when a mutation does not cause differences in plasticity or tolerance breadth between genotypes [24]. In contrast, a concave selection reaction norm with c ≠ 0 leads to a displaced non-central chi-square distribution (with one degree of freedom) for *s*. This distribution may be highly asymmetric if its non-centrality parameter is small, that is, if the linear term is small relative to the quadratic term in Eq (2). This occurs when the average environment is close to the optimum environment for selection (Fig 1C, 1D and 1F).

## Supporting information

S1 Fig Selection reaction norm (*s*) fitted during on transfer depends on past (E_*t-1*_) and current (E_*t*_) environments. Selection coefficients and their standard errors (dots and error bars) were computed from the realized logit allele frequencies estimated at two successive transfers.
(TIF)

S2 Fig Difference between growth rate in isolation of strain C and A (x axis) does not correlate with selection coefficients estimated between two successive transfers (y axis). Growth rates estimates were extracted from Rescan et al. (2020), and selection coefficients were computed from the realized logit allele frequencies estimated between two successive transfers. Colors correspond to the salinity before transfer, from blue (0 M) to black (4.8 M).
(TIF)

S3 Fig Calibration at the ITS2 (A) and the chloroplast locus (A). Observed frequency of allele C is plotted against expected C frequency (circles). The reference strains used to prepare calibration cultures were contaminated by *Dunaliella viridis*. They did not appear in the chloroplast data were primers were designed especially for our strains, but represented more than 50% of the ITS2 sequences (triangles in the inset in a., which represents the frequencies of all strains: A, C, and *D*. *viridis*). However, this contamination of the reference cultures did not affect the correlation between expected and observed frequencies when discounting *D*. *viridis* alleles, which were not present in our experiment.
(TIF)

S1 Table Effect of past (*E*_t-1_) and current (*E*_t_) environment on selection (Estimates, Standard errors and P-values from Wald test). (DOCX)

S2 Table Sampling plan summary. DNA from all populations with population size > 10^3^ cells.mL-1 have been extracted (E in column ‘status’). Successful amplification of both the chloroplast and the ITS2 were sequenced (status S). Samples with a number of reads > 0 and no contamination suspicion at one locus at least were analyzed (status A).
(XLSX)

S1 Appendix Investigation on how our experimental set up consisting of successive transfers of 3 and 4 days, with constant salinity in between transfers, impacts the stochastic dynamics of allelic frequencies. (PDF)
