# Supplementary figures and images for: Predicting population genetic change in an autocorrelated random environment: Insights from a large automated experiment
Source: PLoS Genet. 2021 Jun 23;17(6):e1009611. doi: 10.1371/journal.pgen.1009611 (PMC8259966; doi:10.1371/journal.pgen.1009611)

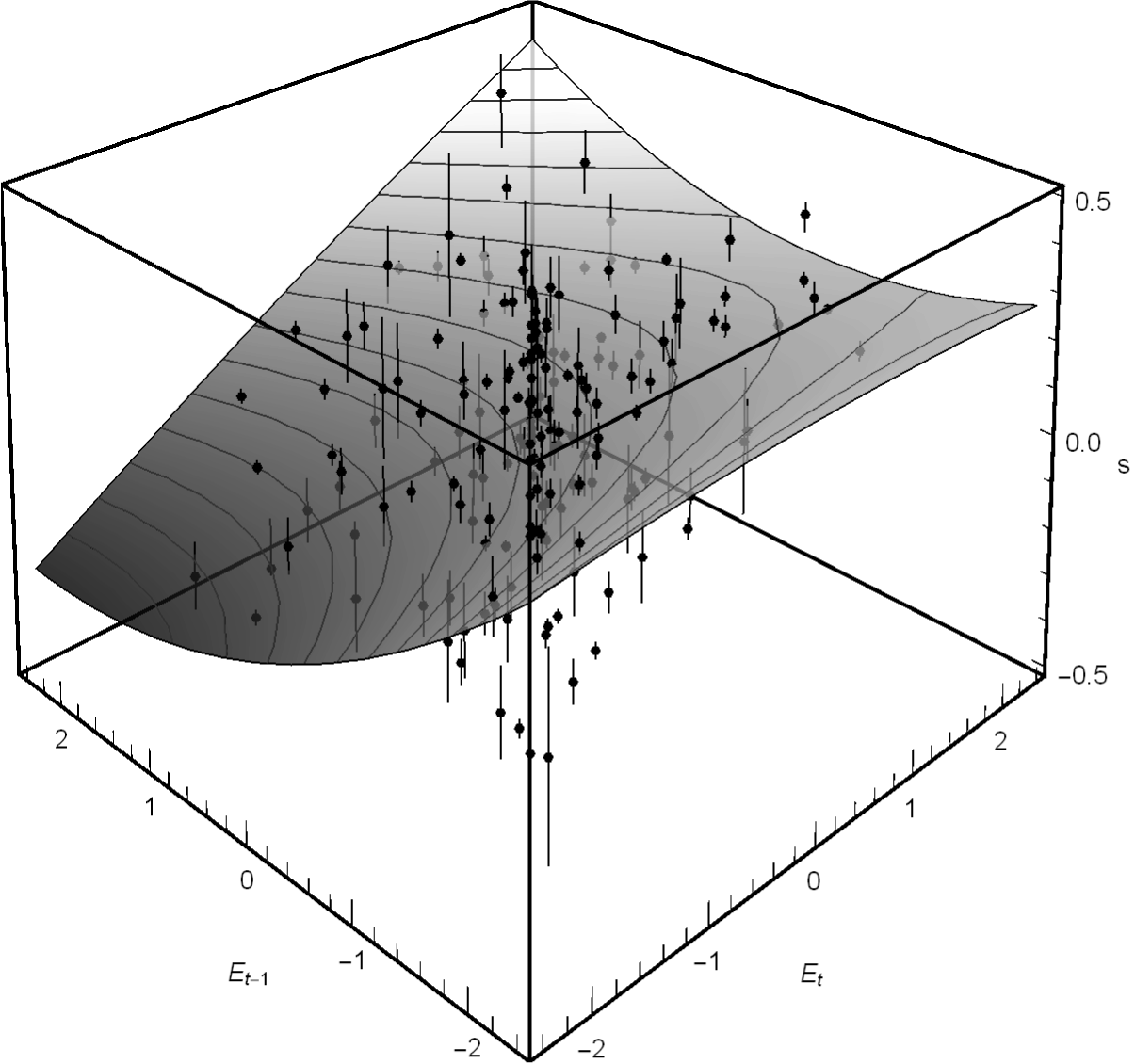

Supplement: S1 Fig — Selection coefficients and their standard errors (dots and error bars) were computed from the realized logit allele frequencies estimated at two successive transfers. (TIF) [file pgen.1009611.s001.tif]

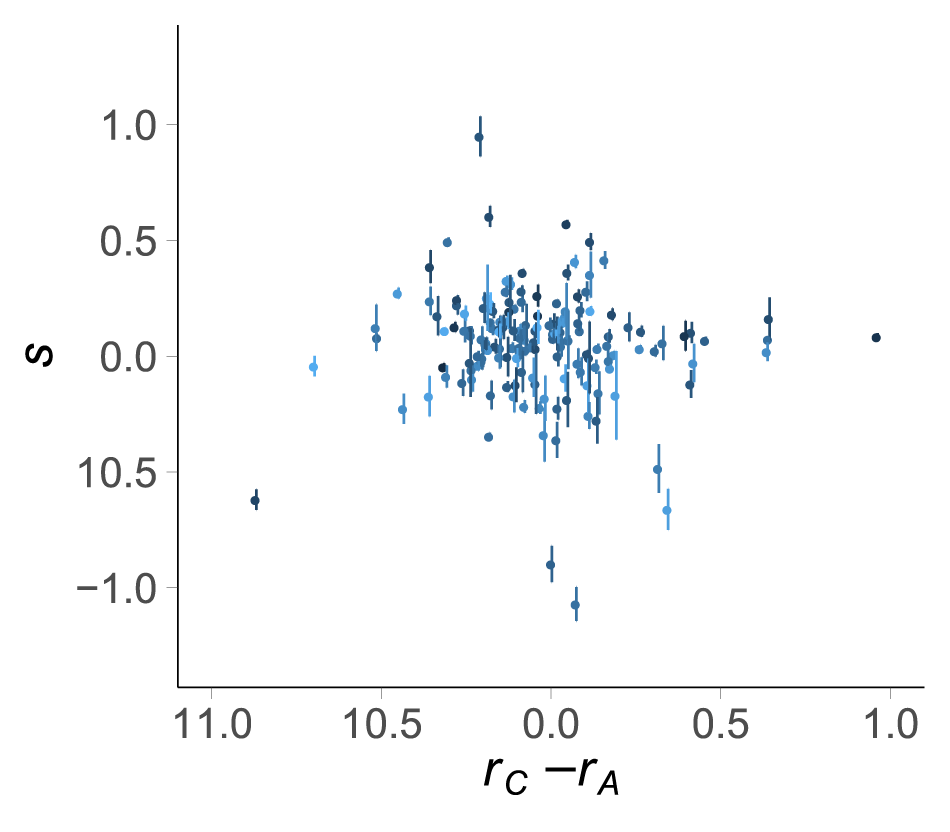

Supplement: S2 Fig — Growth rates estimates were extracted from Rescan et al. (2020), and selection coefficients were computed from the realized logit allele frequencies estimated between two successive transfers. Colors correspond to the salinity before transfer, from blue (0 M) to black (4.8 M). (TIF) [file pgen.1009611.s002.tif]

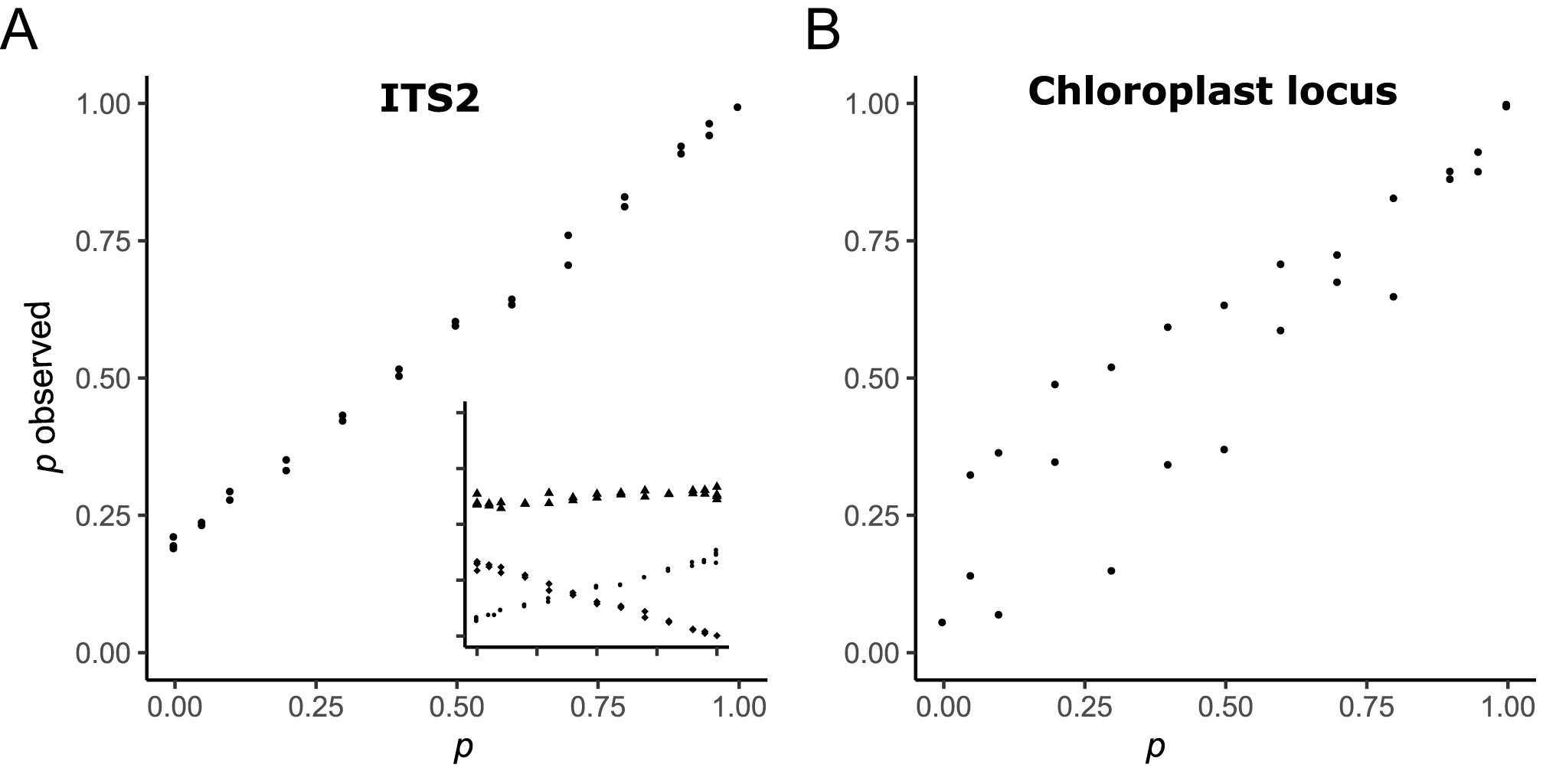

Supplement: S3 Fig — Calibration at the ITS2 (A) and the chloroplast locus (A). Observed frequency of allele C is plotted against expected C frequency (circles). The reference strains used to prepare calibration cultures were contaminated by Dunaliella viridis. They did not appear in the chloroplast data were primers were designed especially for our strains, but represented more than 50% of the ITS2 sequences (triangles in the inset in a., which represents the frequencies of all strains: A, C, and D. viridis). However, this contamination of the reference cultures did not affect the correlation between expected and observed frequencies when discounting D. viridis alleles, which were not present in our experiment. (TIF) [file pgen.1009611.s003.tif]
