# Supplementary material for: Predicting population genetic change in an autocorrelated random environment: Insights from a large automated experiment
Source: PLoS Genet. 2021 Jun 23;17(6):e1009611. doi: 10.1371/journal.pgen.1009611 (PMC8259966; doi:10.1371/journal.pgen.1009611)
Supplement: S1 Table — (DOCX) [file pgen.1009611.s004.docx]

|  | *Estimate* | *Std. Error* | *Pr(>\|W)* |
| --- | --- | --- | --- |
| 1 | 0.0437 | 0.02451 | 7.48E-02 |
| $E_{t}$ | 0.0775 | 0.0198 | 9.33E-05 |
| $E_{t}^{2}$ | -0.004528 | 0.0174 | 7.95E-01 |
| $E_{t-1}$ | -0.0400 | 0.0196 | 4.12E-02 |
| $E_{t-1}^{2}$ | 0.0267 | 0.0181 | 1.39E-01 |
| $E_{t-1}*E_{t}$ | 0.0312 | 0.0181 | 8.50E-02 |
| $\sigma_{s}^{2}$ | 0.00521 | 0.000594 | 1.80E-18 |
