# Supplementary material for: Predicting population genetic change in an autocorrelated random environment: Insights from a large automated experiment
Source: PLoS Genet. 2021 Jun 23;17(6):e1009611. doi: 10.1371/journal.pgen.1009611 (PMC8259966; doi:10.1371/journal.pgen.1009611)
Supplement: S1 Appendix — (PDF) [file pgen.1009611.s006.pdf]

Here, we investigate how our experimental set up consisting of successive transfers of 3 and 4 days, with constant salinity in between transfers, impacts the stochastic dynamics of allelic frequencies.

Between transfers $T - 1$ and T, each population i of a fluctuating salinity treatment grows in a specific salinity $E_{i,T}$, which remains constant until the next transfer. Assuming frequency- and density-independent selection, the selection coefficient $s_{i,T}$ is also constant for each line in between two transfers (and more generally, even when the selection coefficient does vary, we can only estimate an average selection coefficient $s_{i,T}$ over the interval between two transfers). The logit-transformed allelic frequency in line i just before the next transfer is thus (from eq. 10 in the main text):

$$\Psi_{i,T} = \Psi_{i,T-1} + s_{i,T}\Delta_{T-1} \qquad \text{S1}$$

where Δ_{T-1} is the time interval between transfers $T - 1$ and T. Assuming a normal distribution of distribution coefficients (with mean $\bar{s}$ and variance σ_s^2), the distribution of allelic frequencies in the n lines under the stochastic treatment is also normal, with mean

$$\overline{\Psi_T} = \frac{1}{i}\sum_{i=1}^{n}\Psi_{i,T} = \overline{\Psi_{T-1}} + \bar{s}\Delta_{T-1} \qquad \text{S2}$$

and variance:

$$Var(\Psi_T) = Var(\Psi_{T-1}) + Var\left(s_{i,T}\Delta_{T-1}\right) = Var(\Psi_{T-1}) + \Delta_{T-1}^2\sigma_s^2 \qquad \text{S3}$$

Note that the variance does not increase linearly, but quadratically with the time interval between transfers Δ_{T-1}.

After the next transfer, the mean and variance of logit allele frequency are:

$$\overline{\Psi_{T+1}} = \overline{\Psi_{T-1}} + \bar{s}(\Delta_{T-1} + \Delta_T)$$

$$Var(\Psi_{T+1}) = Var(\Psi_{T-1}) + \sigma_s^2(\Delta_{T-1}^2 + \Delta_T^2 + 2\Delta_{T-1}\Delta_T\rho_s) \qquad \text{S4}$$

where ρ_s is the correlation of selection coefficients between subsequent salinities in the time series. In our experimental set up, we had bi-weekly transfers, such that $\Delta_{T-1} = 3$ (or 4) and $\Delta_T = 4$ (or 3), leading to:

$$\overline{\Psi_{T+1}} = \overline{\Psi_{T-1}} + 7\bar{s}$$

$$Var(\Psi_T) = Var(\Psi_{T-1}) + \sigma_s^2(25 + 24\rho_s) \qquad \text{S5}$$

In the logistic regression described in the main text, we infer the mean and variance of selection as the slopes of temporal changes in the mean and variance of logit allelic frequencies, with unit one day. After two transfers, (from $T-1$ to $T+1$), we thus estimated the mean and variance of selection as:

$$\overline{\Psi_{T+1}} = \overline{\Psi_{T-1}} + 7\beta_1$$

$$Var(\Psi_T) = Var(\Psi_{T-1}) + 7\beta_2 \qquad \text{S6}$$

For the mean selection coefficient, comparing eqs. S5 and S6, we directly have $\bar{s} = \beta_1$. For the variance of selection coefficients, neglecting autocorrelation ρ_s for simplicity, we get $\beta_2 \approx \frac{25}{7}\sigma_s^2 \approx 3.57\sigma_s^2$. This means that because of our experimental set up where salinity remains constant in between transfers, the variance of logit frequency increases faster with time than in a true discrete-time or continuous-time stochastic set up with the same duration. Therefore, to

directly estimate σ_s^2 in our logistic regression framework, we multiply the coefficient in the regression for the variance by $25/7$ in eq. (1).
